# Surgery

**Published:** 1906-02-24

**Authors:** 


### SURGERY.

**The Pathology and Prevention of Secondary Parotitis.**—Inflammation of the parotid gland, which generally runs on to suppuration sometimes complicates the course of certain diseases. These may be grouped under three heads: (1) acute diseases—namely, typhus, enteric, scarlet fever, diphtheria; (2) chronic diseases—namely, diabetes, stomatitis, and general paralysis of the insane; (3) abdominal, and especially pelvic operations. An abscess is formed in the parotid gland, and this may, if unrelieved, prove fatal, or may give a fatal turn to the serious illness which it complicates. The abscess may leak into the mouth through the parotid duct; it may burst, externally, into the mouth, into the external auditory meatus, into the joint of the lower jaw, or into one of the great blood-vessels of the neck. The explanations of this secondary parotitis have been many and various. The *sympathetic theory* has been commonly accepted in this country. Because mumps or primary parotitis often affects the ovaries, testes, or mammæ at the same time as the parotid gland, it has been assumed that there is a sympathetic relationship between these structures, and that operation on the ovaries produces secondary parotitis by " sympathy." But apart from the fact that this really constitutes no explanation, it is evident that as secondary parotitis often arises in operations which do not involve the genital organs, the theory will not cover the facts. The *pyœmic theory* explained the facts by supposing that septic material was taken from the area of original disease and lodged in the vessels of the parotid. But against this is the fact that in many cases the original disease is not septic at all—for example, a simple ovariotomy. Also that post-mortem examination proves that the vessels are not the seat of septic thrombi, and if the process was pyæmic, then many abscesses would be found in the lungs and elsewhere, as in other cases of pyæmia. Bucknall [1] brings forward some convincing evidence in favour of Hanan and Pilliet's theory that secondary parotitis is always due to infection of the gland through the ducts. He relates the cases of six patients who died with secondary parotitis. Two had post-operative peritonitis, one had uræmia, two had typhoid, and one diabetic coma. By a bacteriological and histological examination it was proved in every case (1) that the ducts were filled with debris full of micro-organisms; (2) that the inflammation of the gland always started from the ducts whilst the vessels were free; (3) that the same micro-organisms were present in the gland, the pus from the abscess cavity, and the oral mucous membrane in the vicinity of the duct orifice. This micro-organism, which was generally a staphylococcus, was not always the same as that causing the original disease. Now Claisse and Duplay have shown by experiments on animals that it is impossible to produce parotitis by merely smearing the duct orifice with ordinary bacteria. But that if the bacteria be of exalted virulence, if the duct is tied after infection so as to stop salivary flow, if the secretion of saliva be arrested by opium, or if the animal is weak and ill from starvation, then under any of these circumstances suppuration will result from infection of the parotid duct. Now it is evident one or more of these circumstances occur in every one of the diseases and operations likely to be followed by parotitis. Thus in fevers the patient breathes with the open mouth, loses moisture by excessive sweating, and takes no solid food to act as a salivary stimulus. For these reasons the salivary flow becomes almost stagnant, and bacteria accumulate in the mouth. Further, there is an absence of the mechanical cleaning the mouth by chewing. After abdominal operations, the withholding of food and of liquids for a long time produces the same conditions which are often increased by the administration of opium. The importance of these facts lies in the possibility of preventing the condition. Thus the mouth should be cleaned often, especially after vomiting, water not unnecessarily withheld, food given as soon as possible, and the bowels opened. There is probably an analogy between pancreatitis and parotitis as regards their method of origin. And occasionally a very acute case of parotitis occurs similar to those of acute pancreatitis. Carr [2] relates such a case in a patient of 79, who was the subject of gouty eczema and had slight albuminuria. Rapid unilateral swelling of the parotid was accompanied by fever and great pain. Definite fluctuation never occurred, but on the fifth day pus began to ooze from the ear. On the sixth day an incision was made, and the whole gland found to be broken up into necrotic shreds and infiltrated with pus. He died the same day.

**Removal of a Hat Pin from the Lung.**—Russell [3] relates a most remarkable and quite unique case of this operation. The patient was a boy of 12, who five weeks previously had swallowed a three-inch long hat pin. That is to say, he was holding the pin in his mouth, head backwards, and was suddenly jerked and the pin disappeared. An *x*-ray photograph showed the pin to be lying in the lower part of the left lung in the position of the termination of the bronchus. Six inches of the left eighth rib were removed, air was slowly admitted to the pleural cavity, the lower lobe of the lung was grasped, and the head of the pin could be felt through it. It was cut down on and removed by sinus forceps. A small abscess cavity had begun to form round the head of the pin. The boy made a good recovery.

[1] Lancet, Oct. 2, 1905. [2] Lancet, Sept. 16, 1905. [3] Lancet, Sept. 9, 1905.

